# Asymptomatic hyperuricemia and coronary artery disease in elderly patients without comorbidities

**DOI:** 10.18632/oncotarget.21079

**Published:** 2017-09-19

**Authors:** Junnan Wu, Guangtao Lei, Xiao Wang, Yuezhong Tang, Huan Cheng, Guihua Jian, Xianfeng Wu, Niansong Wang

**Affiliations:** ^1^ Department of Nephrology, Affiliated Sixth People's Hospital, Shanghai Jiao Tong University, Shanghai, China; ^2^ Department of Cardiology, The Second Affiliated Hospital of Nanchang University, Nanchang, China; ^3^ Department of Endocrinology, Affiliated Sixth People's Hospital, Shanghai Jiao Tong University, Shanghai, China; ^4^ Kangjian Community Health Center, Xuhui District, Shanghai, China

**Keywords:** comorbidities, coronary artery disease, elderly, hyperuricemia, serum uric acid

## Abstract

Because many subjects with hyperuricemia have comorbidities, it can be difficult to differentiate the role of hyperuricemia from that of other comorbidities of coronary artery disease (CAD). Subjects aged ≥ 65 years were enrolled in the study and were available at enrollment and at 5-year follow-up. Subjects were excluded if they were overweight or obese, hypertensive, diabetic, hyperlipidemic, had a pre-existing cardiovascular disease, a history of gout or hyperuricemia on medications, or chronic kidney disease as estimated by a glomerular filtration rate (eGFR) < 60 mL/min per 1.73 m^2^. We used Poisson regression to estimate the hazard ratio (HR) for incident CAD events between hyperuricemic (> 7 mg/dL in men and ≥ 6 mg/dL in women) and normouricemic subjects. A total of 2,142 subjects without comorbidities (mean age of 70.7 ± 5.9 years, 1,194 men) were followed for 57.4 ± 8.9 months. Hyperuricemia was associated with an increased cumulative incidence of incident CAD events (15.0% versus 8.8%, *P* < 0.001). After adjusting for confounding factors, hyperuricemia independently predicted the risk of incident CAD events (HR=1.71, 95% CI 1.26–2.34). In conclusion, asymptomatic hyperuricemia is a valuable biomarker for predicting the development of incident CAD events.

## INTRODUCTION

Hyperuricemia is known to be associated with cardiovascular disease (CVD), such as coronary artery disease (CAD), stroke and hypertension [1], but the role of serum uric acid (SUA) as an independent risk factor for CVD remains unclear. Many epidemiologic studies have shown that hyperuricemia is frequently noted in patients either with CVD or at a high risk of CVD, such as hypertension, CAD, stroke, heart failure, metabolic syndrome, and peripheral vascular disease [2–7]. Most investigators reasoned that SUA may become passively elevated due to the effects of insulin resistance, renal vasoconstriction, and reduced estimated glomerular filtration rate (eGFR) to reduce uric acid excretion by the kidneys [8]. However, experimental studies have suggested that SUA may have an independent modulatory or causal role in these conditions [9–11]. Consistent with these findings, an elevated SUA has been consistently found to predict the development of CAD [1]. Unfortunately, because many of the subjects with hyperuricemia have comorbidities, it can be difficult to differentiate the role of SUA from the coexistence of the other comorbid conditions. Although multivariable analysis can be used to control for these other conditions, multivariable analysis can be misleading if the associated risk factors are causally linked [12]. The limitations associated with multivariable analysis as a means for determining causation are well known [8, 13, 14].

CAD is the leading cause of mortality in the elderly, and more than 80% of the CAD mortalities occur in persons older than 65 years of age [15, 16]. The elderly are more afflicted with chronic diseases or comorbidities, which may interfere with CAD incidence. To date, there is a paucity of studies on CAD incidence in elderly subjects without comorbidities. Therefore, an alternative approach is to limit a prospective study to elderly subjects with hyperuricemia who do not have any other cardiovascular, metabolic, or renal risk factors. We investigated whether asymptomatic hyperuricemic elderly subjects without comorbidities have an increased risk for incident CAD events.

## RESULTS

### Patient characteristics

**Figure 1:**
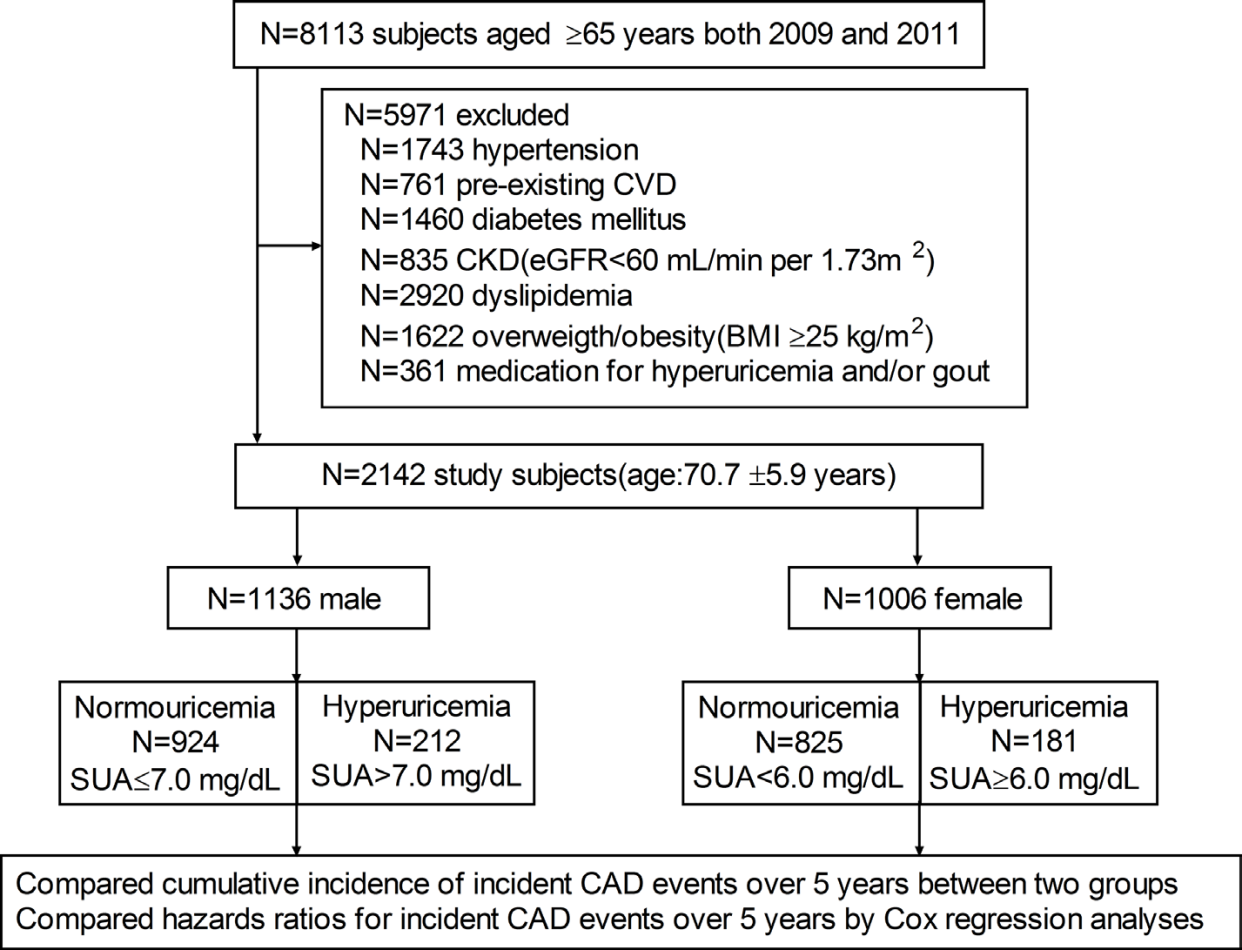
Flow diagram of study enrollment N = number of subjects; CVD = cardiovascular disease; CKD = chronic kidney disease; eGFR = estimated glomerular filtration rate; BMI = body mass index; SUA = serum uric acid.

There were 8,113 subjects aged ≥ 65 years who underwent annual medical examination at the Kangjian Community Health Center of Shanghai in 2009 and 2011. Of the 8,113 subjects, we excluded 5,971 subjects with comorbidities at baseline: 1,743 with hypertension, 761 with pre-existing CVD, 1,460 with diabetes mellitus (DM), 835 with chronic kidney disease (CKD), 2,920 with hyperlipidemia, 1,622 who were overweight/obese and 361 subjects with hyperuricemia or gout who were on medication (Figure 1). Thus, 2,142 patients were enrolled in the study (mean age of 70.7 ± 5.9 years, 1194 men) with a mean follow-up of 57.4 ± 8.9 months.

Baseline demographic and clinical characteristics of the cohort are given in Table 1. Hyperuricemic subjects showed mild but significant differences from normouricemic subjects for systolic blood pressure (BP) and estimated GFR, despite both groups having values within the normal range. Compared with normouricemic subjects, hyperuricemic subjects more frequently had smoking and drinking habits, as well as presented with higher hemoglobin, total protein, albumin, serum creatinine, total-cholesterol, triglycerides, and low density lipoprotein (LDL), but a lower high density lipoprotein (HDL) and eGFR than total subjects. Similar differences of baseline characteristics were also found between male and female subjects, except for systolic BP.

**Table 1:** Baseline characteristics of the study subjects

	Total			Male sex			Female sex		
	Normouricemia (*n* = 1749)	Hyperuricemia (*n* = 393)	*p*-value	Normouricemia (*n* = 954)	Hyperuricemia (*n* = 238)	*p*-value	Normouricemia (*n* = 795)	Hyperuricemia (*n* = 155)	*p*-value
Number of subjects	1749	393		971	223		778	170	
Male sex	55.5%	56.7%	0.694						
Age, years	70.7 ± 6.0	70.7 ± 5.7	0.924	71.0 ± 6.0	70.5 ± 5.8	0.331	70.3 ± 6.0	70.9 ± 5.7	0.222
BMI, kg/m^2^	22.5 ± 1.6	22.3 ± 1.6	0.129	22.4 ± 1.6	22.3 ± 1.7	0.670	22.5 ± 1.6	22.3 ± 1.6	0.066
Systolic BP, mmHg	120.5 ± 10.8	121.8 ± 9.0	0.032	120.2 ± 10.9	121.5 ± 8.9	0.095	120.9 ± 10.8	122.1 ± 9.2	0.174
Diastolic BP, mmHg	73.6 ± 7.5	73.9 ± 7.2	0.524	73.3 ± 7.6	74.2 ± 7.2	0.141	74.0 ± 7.4	73.5 ± 7.1	0.473
Pulse rate, bpm	73.1 ± 10.8	73.9 ± 9.6	0.169	73.4 ± 11.0	73.6 ± 8.9	0.817	72.8 ± 10.7	74.4 ± 10.4	0.070
Current smoking	27.4%	59.8%	< 0.001	55.4%	61.4%	< 0.001	13.9%	25.9%	< 0.001
Drinking habits	36.7%	68.2%	< 0.001	60.7%	70.4%	< 0.001	14.9%	28.2%	< 0.001
Hemoglobin (g/dL)	13.4 ± 1.3	14.3 ± 1.2	< 0.001	13.5 ± 1.3	14.3 ± 1.2	< 0.001	13.3 ± 1.2	14.2 ± 1.2	< 0.001
Total protein, g/dL	7.2 ± 0.5	7.4 ± 0.6	< 0.001	7.2 ± 0.5	7.3 ± 0.6	< 0.001	7.1 ± 0.5	7.4 ± 0.6	< 0.001
Albumin, g/dL	4.3 ± 0.4	4.4 ± 0.4	< 0.001	4.3 ± 0.4	4.4 ± 0.4	0.092	4.3 ± 0.4	4.5 ± 0.4	< 0.001
Serum creatinine, mg/dL	0.8 ± 0.1	0.9 ± 0.1	< 0.001	0.8 ± 0.1	0.9 ± 0.1	< 0.001	0.7 ± 0.1	0.8 ± 0.1	< 0.001
eGFR, ml/min per 1.73m^2^	86.1 ± 14.7	81.4 ± 12.5	< 0.001	85.9 ± 13.7	81.1 ± 12.3	< 0.001	86.5 ± 13.9	82.8 ± 12.6	< 0.001
SUA, mg/dL	5.0 ± 0.9	8.6 ± 1.6	< 0.001	5.1 ± 0.9	8.6 ± 1.8	< 0.001	4.8 ± 0.8	8.6 ± 1.3	< 0.001
Triglycerides (mmol/L)	1.5 ± 0.9	1.8 ± 1.0	< 0.001	1.6 ± 0.9	1.8 ± 1.0	< 0.001	1.5 ± 0.8	1.7 ± 1.2	< 0.001
Total-cholesterol (mmol/L)	5.1 ± 1.1	5.3 ± 1.2	< 0.001	5.1 ± 1.1	5.3 ± 1.1	0.018	5.1 ± 1.0	5.3 ± 1.4	< 0.001
LDL (mmol/L)	3.3 ± 0.9	3.5 ± 0.9	< 0.001	3.2 ± 0.9	3.5 ± 0.9	< 0.001	3.3 ± 0.8	3.5 ± 1.0	< 0.001
HDL (mmol/L)	1.7 ± 0.5	1.5 ± 0.4	< 0.001	1.7 ± 0.5	1.4 ± 0.4	< 0.001	1.7 ± 0.5	1.5 ± 0.4	< 0.001

BMI = body mass index; BP = blood pressure; eGFR = estimated glomerular filtration rate; SUA = serum uric acid; LDL = low density lipoprotein; HDL = high density lipoprotein.

### Cumulative incidence of incident CAD between 2011 and 2016

**Figure 2:**
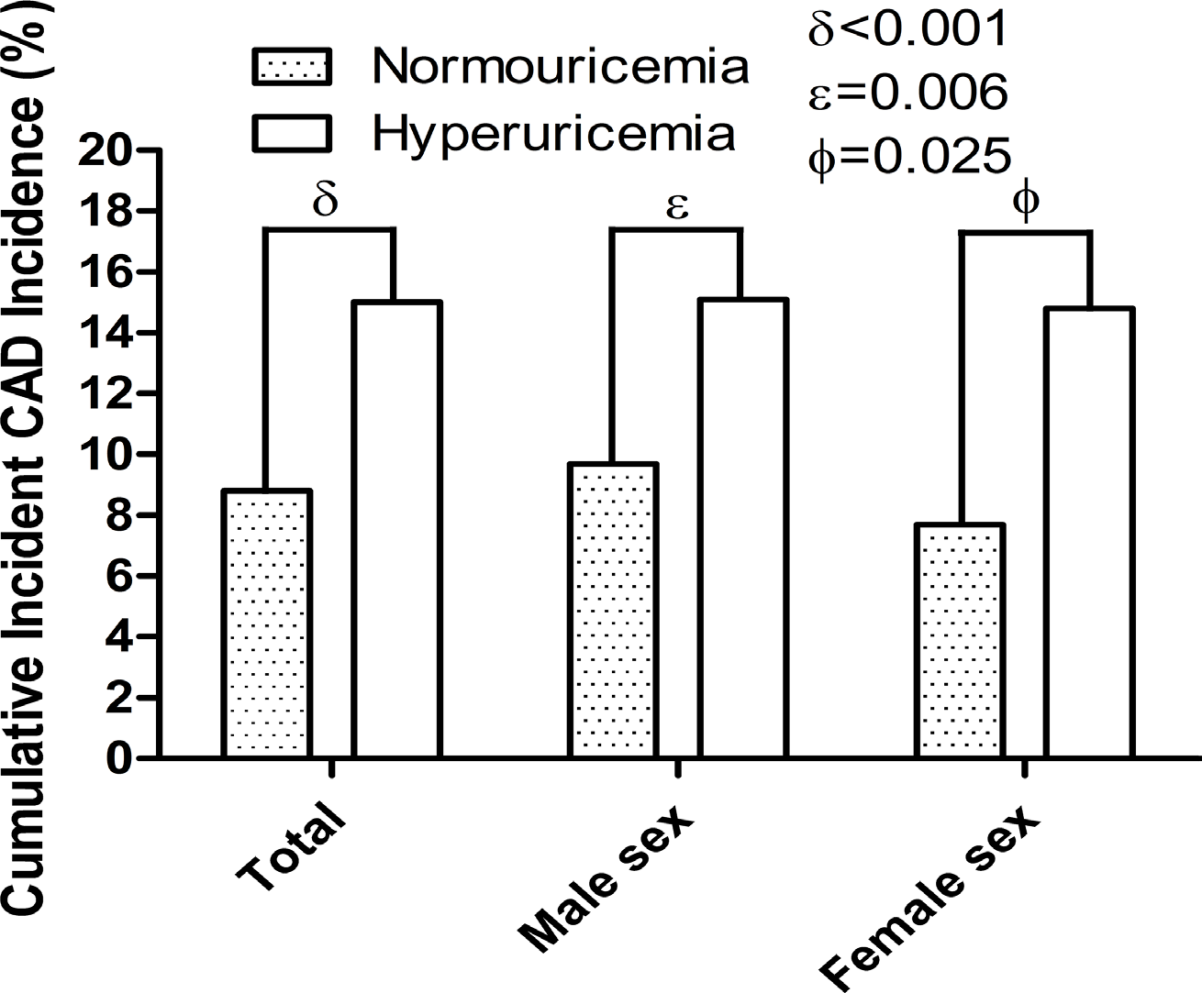
The 5-year cumulative incidence of incident CAD events between hyperuricemia and normouricemia Hyperuricemia had higher 5-year cumulative incidence of incident CAD events compared to normouricemia in total population (*p* < 0.001), male (*p* = 0.006) and (*p* = 0.025) female subjects. CAD = coronary artery disease.

During the follow-up, 407 (19.0%) deaths occurred, including 218 (10.2%) CVD deaths and 63 (2.9%) loss of follow-up cases in the study population. The number of major CVD adverse events during each year are shown in Table 2. Subject characteristics at the end of the study are shown in Table 3. A total of 225 CAD events occurred in this study population, of which 213 (9.9%) were incident CAD events. We compared the cumulative incidence of incident CAD events over 5 years for subjects with hyperuricemia compared with those with normouricemia (Figure 2). In the study population, hyperuricemic subjects showed significantly higher cumulative incidence of incident CAD than normouricemic subjects (15.0% versus 8.8%, hazard ratio [HR] = 1.83, 95% confidence index [CI] 1.33–2.53, *p* < 0.001).

**Table 2:** Numbers of major CVD events during the follow-up

	Total		Male sex		Female sex	
	Normouricemia (*n* = 1749)	Hyperuricemia (*n* = 393)	Normouricemia (*n* = 954)	Hyperuricemia (*n* = 238)	Normouricemia (*n* = 795)	Hyperuricemia (*n* = 155)
At 1-year						
CAD	15	5	10	3	5	2
Congestive heart failure	4	2	2	1	2	1
Stroke	6	2	3	1	3	1
Peripheral vascular disease	0	1	0	0	0	1
At 2-year						
CAD	25	8	16	5	9	3
Congestive heart failure	6	3	3	2	3	1
Stroke	11	2	6	1	5	1
Peripheral vascular disease	2	2	1	1	1	1
At 3-year						
CAD	31	13	18	8	13	5
Congestive heart failure	15	5	9	3	6	2
Stroke	13	6	8	4	5	2
peripheral vascular disease	8	3	5	2	3	1
At 4-year						
CAD	39	16	22	9	17	6
Congestive heart failure	16	6	10	4	6	2
Stroke	18	9	11	6	7	3
Peripheral vascular disease	11	5	7	4	4	1
At 5-year						
CAD	44	17	26	11	18	7
Congestive heart failure	21	7	13	5	8	2
Stroke	23	13	13	8	10	5
Peripheral vascular disease	12	6	7	4	5	2
Cumulative Numbers of major CVD events over 5 years						
CAD	154 (8.8%)	59 (15.0%)	92 (9.7 %)	36 (15.1%)	62 (7.8%)	23 (14.8%)
Congestive heart failure	62 (3.6%)	23 (5.9%)	37 (3.9%)	15 (6.1%)	25 (3.2%)	8 (5.2%)
Stroke	73 (4.2%)	32 (8.1%)	41 (4.3%)	20 (8.2%)	30 (3.8%)	12 (7.7%)
Peripheral vascular disease	33 (1.9%)	17 (4.6%)	20 (2.1%)	11 (4.6%)	13 (1.6%)	6 (3.7%)

CAD = coronary artery disease; CVD = cardiovascular disease.

**Table 3:** Subject characteristics at the end of study

	Total			Male sex			Female sex		
	Normouricemia	Hyperuricemia	*p*-value	Normouricemia	Hyperuricemia	*p*-value	Normouricemia	Hyperuricemia	*p*-value
Age, years	73.7 ± 6.1	73.5 ± 5.8	0.637	73.9 ± 6.1	73.6 ± 5.9	0.256	73.5 ± 6.0	73.2 ± 5.8	0.209
BMI, kg/m^2^	22.1 ± 1.5	22.2 ± 1.6	0.324	22.3 ± 1.5	22.6 ± 1.6	0.431	22.0 ± 1.5	22.1 ± 1.5	0.143
Systolic BP, mmHg	122.4 ± 11.1	123.7 ± 9.8	0.029	122.7 ± 11.0	123.8 ± 9.1	0.137	122.1 ± 10.7	122.9 ± 10.3	0.168
Diastolic BP, mmHg	75.8 ± 7.4	75.7 ± 7.3	0.631	75.9 ± 7.5	76.0 ± 7.4	0.346	75.1 ± 7.5	75.6 ± 7.2	0.516
Pulse rate, bpm	74.5 ± 10.2	74.8 ± 9.8	0.241	74.8 ± 10.8	75.0 ± 9.7	0.673	74.5 ± 10.0	74.3 ± 10.4	0.238
Current smoking	27.7%	60.3%	< 0.001	55.9%	61.8%	< 0.001	14.3%	26.1%	< 0.001
Drinking habits	36.9%	68.6%	< 0.001	60.9%	70.7%	< 0.001	15.2%	28.7%	< 0.001
Hemoglobin (g/dL)	12.9 ± 1.2	13.8 ± 1.1	< 0.001	13.1 ± 1.2	13.9 ± 1.2	< 0.001	12.7 ± 1.1	13.6 ± 1.2	< 0.001
Total protein, g/dL	7.1 ± 0.5	7.3 ± 0.5	< 0.001	7.2 ± 0.5	7.4 ± 0.6	< 0.001	7.0 ± 0.5	7.2 ± 0.6	< 0.001
Albumin, g/dL	4.2 ± 0.4	4.3 ± 0.4	< 0.001	4.2 ± 0.4	4.3 ± 0.4	0.092	4.2 ± 0.4	4.3 ± 0.4	< 0.001
Serum creatinine, mg/dL	0.9 ± 0.1	1.0 ± 0.1	< 0.001	0.9 ± 0.1	1.0 ± 0.1	< 0.001	0.8 ± 0.1	0.9 ± 0.1	< 0.001
eGFR, ml/min per 1.73m^2^	81.1 ± 12.3	76.4 ± 11.9	< 0.001	82.3 ± 11.7	79.3 ± 11.8	< 0.001	78.5 ± 11.7	75.3 ± 11.2	< 0.001
SUA, mg/dL	5.3 ± 1.5	9.1 ± 1.7	< 0.001	5.4 ± 1.2	9.2 ± 1.8	< 0.001	5.2 ± 1.1	8.8 ± 1.7	< 0.001
Triglycerides(mmol/L)	1.7 ± 1.0	2.0 ± 1.0	< 0.001	1.8 ± 0.9	2.1 ± 1.0	< 0.001	1.6 ± 0.9	1.9 ± 1.1	< 0.001
Total-cholesterol(mmol/L)	5.3 ± 1.2	5.4 ± 1.2	< 0.001	5.4 ± 1.2	5.5 ± 1.2	0.010	5.2 ± 1.0	5.3 ± 1.2	< 0.001
LDL(mmol/L)	3.4 ± 1.0	3.6 ± 1.0	< 0.001	3.4 ± 1.0	3.6 ± 0.9	< 0.001	3.4 ± 0.9	3.5 ± 1.0	< 0.001
HDL(mmol/L)	1.6 ± 0.6	1.4 ± 0.5	< 0.001	1.5 ± 0.5	1.3 ± 0.5	< 0.001	1.7 ± 0.5	1.5 ± 0.5	< 0.001

BMI = body mass index; BP = blood pressure; eGFR = estimated glomerular filtration rate; SUA = serum uric acid; LDL = low density lipoprotein; HDL = high density lipoprotein.

We also compared the cumulative incidences for incident CAD events between hyperuricemia and normouricemia by sex. In men, hyperuricemic subjects showed significantly higher cumulative incidence of incident CAD events than normouricemic subjects (15.1% versus 9.7%, HR = 1.65, 95% CI 1.29–2.49, *p* = 0.006; Figure 2). In women, hyperuricemic subjects showed significantly higher cumulative incidence of incident CAD events compared with normouricemic subjects (14.8% versus 7.7%, HR = 2.09, 95% CI 1.25–2.51, *p* = 0.025; Figure 2). In the study population, there were no sex differences in the cumulative CAD incidence (HR = 0.90, 95% CI 0.80–1.06,p=0.128). In the hyperuricemia and normouricemia subjects, there were also no sex differences in the cumulative CAD incidence, respectively (HR = 0.98, 95% CI 0.95–1.02, *p* = 0.938, and HR = 0.97, 95% CI 0.95–1.02, *p* = 0.127).

### Association of hyperuricemia and incident CAD events using multivariable adjustments

In the study population, in crude analysis, hyperuricemia became an independent risk factor for incident CAD events (HR = 1.82; 95% CI, 1.36–2.46, Table 4). After adjustment included baseline eGFR, hyperuricemia remained an independent risk factor for incident CAD events (HR = 1.71, 95% CI 1.26–2.34; total, Model 3; Table 4). When analysis was restricted to men, hyperuricemia remained an independent risk factor for incident CAD events (HR=1.81; 95% CI, 1.23–2.77; male sex, Model 3; Table 4). When analysis was restricted to women, hyperuricemia became an independent risk factor for incident CAD events (HR = 1.42; 95% CI, 1.10–1.99; female sex, Model 3; Table 4). In addition, hyperuricemia showed an independent risk factor for incident CAD events after adjusting for confounding factors at the ending of the study (HR=1.73, 95%CI 1.27–2.31; total, Model 3; Table 5).

**Table 4:** Baseline serum uric acid and hyperuricemia as a risk for incident CAD events in the subjects

	Crude			Model 1			Model 2			Model 3		
	HR	95% CI	*p*-value	HR	95% CI	*p*-value	HR	95% CI	*p*-value	HR	95% CI	*p*-value
Total = 2142												
Hyperuricemia	1.82	1.36–2.46	< 0.001	1.85	1.38–2.50	< 0.001	1.84	1.37–2.50	< 0.001	1.71	1.26–2.34	0.001
Per SUA 1mg/dL	1.18	1.11–1.25	< 0.001	1.17	1.10–1.24	< 0.001	1.15	1.10–1.25	0.001	1.12	1.04–1.21	0.001
Male sex = 1194												
Hyperuricemia	1.78	1.21–2.62	0.003	1.84	1.25–2.70	0.002	1.83	1.24–2.70	0.023	1.79	1.26–2.78	0.024
Per SUA 1mg/dL	1.18	1.10–1.27	< 0.001	1.15	1.08–1.29	< 0.001	1.10	1.05–1.31	< 0.001	1.07	1.02–1.20	0.046
Female sex = 948												
Hyperuricemia	1.87	1.16–3.02	0.011	1.85	1.37–2.50	< 0.001	1.84	1.36–2.49	< 0.001	1.41	1.10–1.98	0.009
Per SUA 1mg/dL	1.16	1.10–1.29	< 0.001	1.15	1.08–1.38	< 0.001	1.13	1.07–1.39	0.001	1.11	1.06–1.40	0.003

SUA indicated baseline serum uric acid level.

Model 1: Data adjusted for baseline age, sex, smoking and drinking habits.

Model 2: Data adjusted for baseline age, sex, smoking and drinking habits, baseline BMI, baseline systolic BP and diastolic BP.

Model 3: Data adjusted for baseline age, sex, smoking and drinking habits, baseline BMI, baseline systolic BP and diastolic BP, and baseline eGFR, total-cholesterol, triglycerides, LDL and HDL.

CAD = coronary artery disease; BMI = body mass index; BP = blood pressure; eGFR=estimated glomerular filtration rate; SUA = serum uric acid; LDL = low density lipoprotein; HDL= high density lipoprotein; HR = hazards ratio; C I= confidence interval.

**Table 5:** Serum uric acid* and hyperuricemia as a risk for incident CAD events in the subjects

	Crude			Model 1			Model 2			Model 3		
	HR	95% CI	*p*-value	HR	95% CI	*p*-value	HR	95% CI	*p*-value	HR	95% CI	*p*-value
Total = 2142												
Hyperuricemia	1.84	1.41–2.49	< 0.001	1.81	1.40–2.42	< 0.001	1.77	1.39–2.49	< 0.001	1.73	1.27–2.31	0.001
Per SUA 1mg/dL	1.19	1.12–1.26	< 0.001	1.16	1.10–1.23	< 0.001	1.13	1.11–1.24	0.001	1.11	1.05–1.20	0.001
Male sex = 1194												
Hyperuricemia	1.81	1.31–2.61	< 0.001	1.79	1.33–2.59	< 0.001	1.75	1.30–2.4	< 0.001	1.68	1.13–1.89	0.010
Per SUA 1mg/dL	1.20	1.10–1.26	< 0.001	1.18	1.09–1.25	< 0.001	1.15	1.06–1.29	< 0.001	1.12	1.03–1.24	0.038
Female sex = 948												
Hyperuricemia	1.86	1.25–2.60	0.001	1.84	1.26–2.65	0.002	1.80	1.26–2.66	0.019	1.75	1.24–2.67	0.019
Per SUA 1mg/dL	1.15	1.10–1.27	< 0.001	1.14	1.10–1.28	< 0.001	1.12	1.08–1.29	< 0.001	1.10	1.05–1.30	0.001

*SUA indicated serum uric acid level of ending of study.

Model 1: Data adjusted for age, sex, smoking and drinking habits at the end of study.

Model 2: Data adjusted for baseline age, sex, smoking and drinking habits, baseline BMI, baseline systolic BP and diastolic BP at the end of study.

Model 3: Data adjusted for baseline age, sex, smoking and drinking habits, baseline BMI, baseline systolic BP and diastolic BP, and baseline eGFR, total-cholesterol, triglycerides, LDL and HDL at the end of study.

CAD = coronary artery disease; BMI = body mass index; BP = blood pressure; eGFR = estimated glomerular filtration rate; SUA = serum uric acid; LDL = low density lipoprotein; HDL = high density lipoprotein; HR = hazards ratio; CI = confidence interval.

### Cumulative incident CAD event risk between hyperuricemic and normouricemic subjects

**Figure 3:**
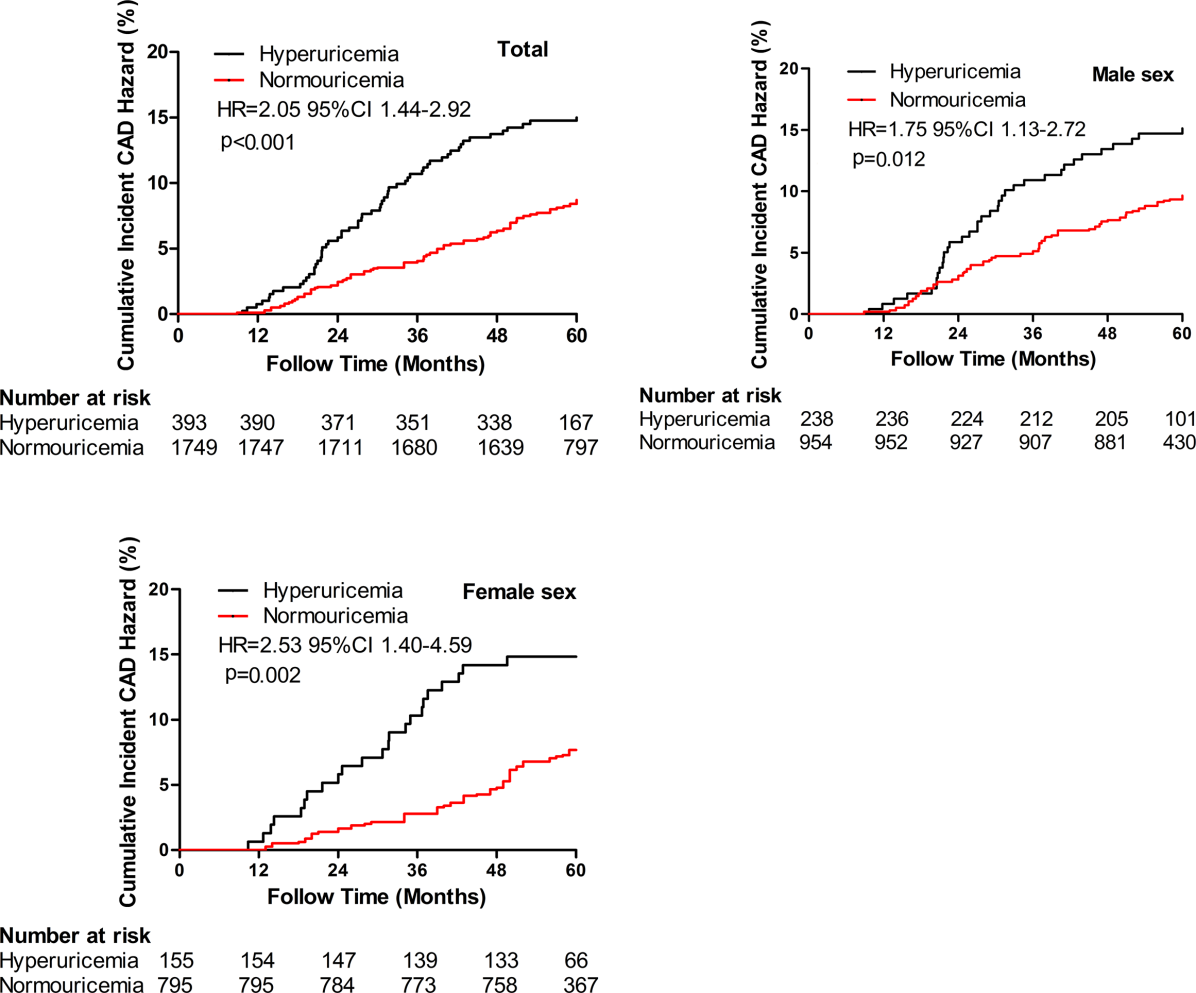
Cumulative incident CAD risk between hyperuricemia and normouricemia Hyperuricemia had higher cumulative incident CAD events risk compared to normouricemia in the study population (*p* < 0.001), male (*p* = 0.012) and female (*p* = 0.002) subjects. CAD = coronary artery disease; HR = hazards ratio; CI = confidence index.

Compared with normouricemic subjects, hyperuricemic subjects were at increased risk of incident CAD events based on Kaplan-Meier curves (Figure 3). In crude analysis of incident CAD event risk, the cumulative risk was significantly higher in the hyperuricemic subjects (HR = 2.05, 95% CI 1.44–2.92). Male hyperuricemic subjects had a higher risk of cumulative incident CAD events than male normouricemic subjects (HR = 1.75, 95% CI 1.13–2.72). In addition, female hyperuricemic subjects also had a higher risk of cumulative incident CAD events than female normouricemic subjects (HR = 2.53, 95% CI 1.40–4.59).

### Subgroup analysis of 5-year cumulative incidences of incident CAD events

**Figure 4:**
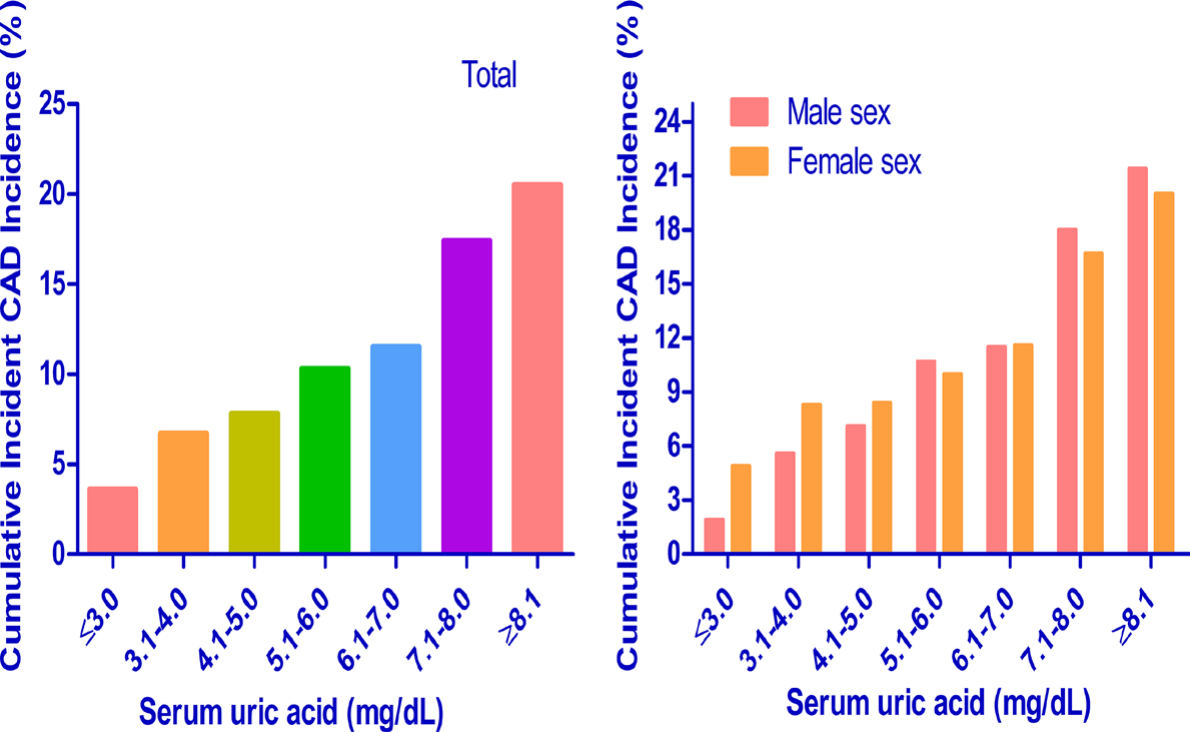
Subgroup analysis of 5-year cumulative incidence of incident CAD events in each serum uric acid level CAD = coronary artery disease.

**Figure 5:**
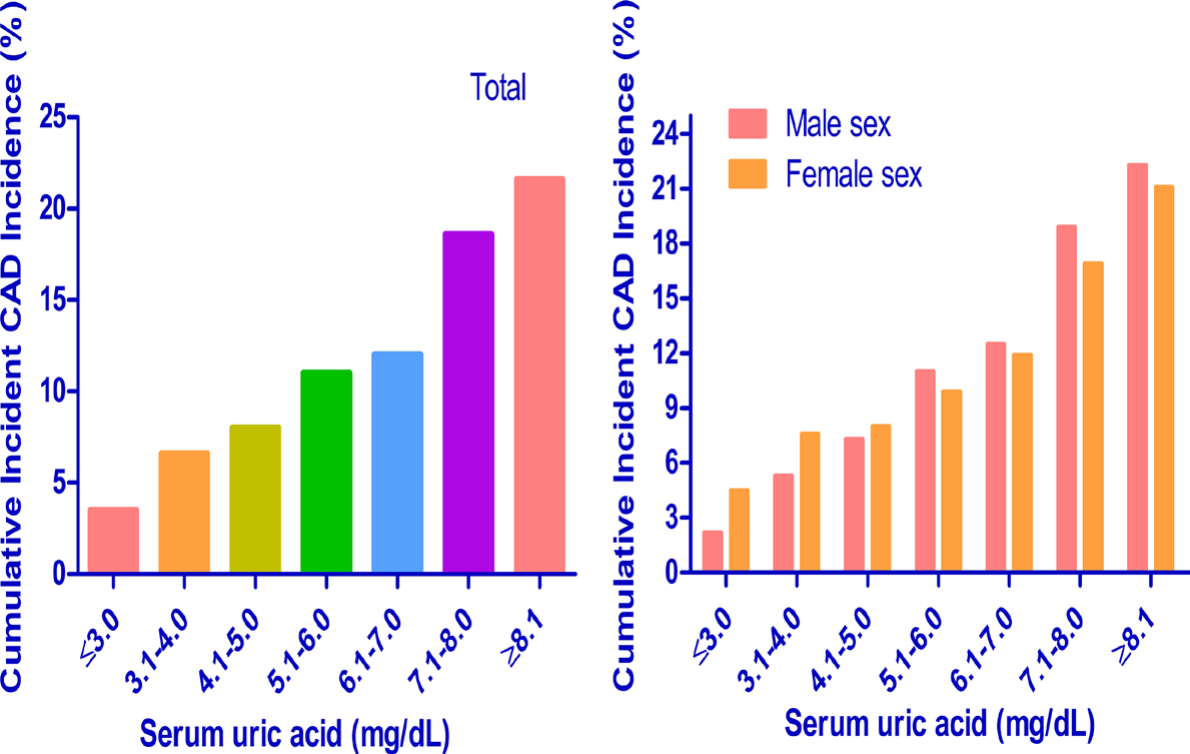
Subgroup analysis of 5-year cumulative incidence of incident CAD events in each serum uric acid level of ending of study CAD = coronary artery disease.

We calculated cumulative incidences of incident CAD events over 5 years for each baseline SUA level: ≤ 3.0, 3.1 to 4.0, 4.1 to 5.0, 5.1 to 6.0, 6.1 to 7.0, 7.1 to 8.0 and ≥ 8.1 mg/dL. In the study population, the 5-year cumulative incidences of incident CAD events were 3.6%, 6.7%, 7.8%, 10.3%, 11.5%, 17.4% and 20.5% (Figure 4). In addition, we calculated cumulative incidences of incident CAD events over 5 years by sex for each SUA level. The 5-year cumulative incidences of incident CAD events were 1.9%, 5.6%, 7.1%, 10.7%, 11.5%, 18.0% and 21.4% in men and 4.9%, 8.3%, 8.4%, 10.0%, 11.6%, 16.7% and 20.0% in women. The results showed that higher baseline SUA had a higher 5-year cumulative incidence of incident CAD events in both men and women. In addition, we found similar cumulative incidences of incident CAD events over 5 years for each SUA level at the ending of study (Figure 5).

## DISCUSSION

Our primary finding was that asymptomatic hyperuricemic subjects without comorbidities had a significant increased (1.82-fold) risk for developing incident CAD events. This study documents that the presence of hyperuricemia is an important biomarker for incident CAD events, but this study does not evaluate whether hyperuricemia has a causal role in the development of this condition.

SUA, the end product of purine metabolism via an enzymatic reaction involving xanthine oxidase, has also been correlated with CAD by several studies [17–19]. However, because of controversial epidemiologic findings and a lack of consistent evidence, it remains unknown whether SUA is an independent and causal risk factor for CAD [7, 20–23]. Several observational studies demonstrated that elevated SUA has a predictive value for CAD risk and that hyperuricemia may be an important causal factor for CAD mortality [20, 24–26]. However, other studies contradict this [19, 22, 27–29]. Many factors may contribute to the conflicting conclusions. Known risk factors, such as age, sex, hypertension, DM, overweight/obesity, CKD and other potential confounding factors, under- or over-estimate the association between hyperuricemia and the risk of related CVD disease. Few studies have used this approach, and most take all subjects with hyperuricemia, of which the majority already carry cardiometabolic comorbidities [30]. Thus, an alternative approach to studying the relationship between hyperuricemia and CAD events is to limit the study population to only subjects with hyperuricemia who do not have other cardiovascular, metabolic or renal risk factors. The importance of our study is that we evaluate subjects with asymptomatic hyperuricemia who lack cardiac and metabolic risk factors to determine if they are still at risk for CVD diseases. Multivariable analysis was also conducted to determine whether the increased risk of asymptomatic hyperuricemia for incident CAD events was independent of age, sex, and smoking and drinking habits (Model 1), or with the addition of baseline body mass index (BMI), systolic baseline BP and diastolic BP (Model 2), or with the addition of baseline eGFR (Model 3). In these models, the presence of hyperuricemia became an independent risk factor for incident CAD events in elderly subjects without comorbidities.

The association between SUA and cardiovascular events in the general population is reported to be stronger in women than in men [31]. Freedman et al. demonstrated a stronger association between increased SUA levels and cardiovascular mortality among healthy women than in healthy men [20]. In the present study, there was no sex difference in the cumulative CAD incidence. The changing association between elderly men and postmenopausal women suggests that there may be an interaction with sex hormones [31].

Previous studies showed that hyperuricemia had a pathogenic role and predictive value in the development of hypertension [9, 32–35]. This finding suggests that a causal link to the development of hypertension is a plausible explanation for the possible increased CAD risk in patients with hyperuricemia [2]. Secondly, the presence of hyperuricemia may contribute to lipid peroxidation and promote the oxidation of low-density lipoprotein cholesterol, which may play a role in the development of atherosclerosis and would also explain its association with CAD events [36–38]. Of note, human atherosclerosis plaques contain more uric acid than normal artery walls, which suggests that SUA may have a direct role in the atherosclerosis process [39]. Thirdly, hyperuricemia may induce endothelial dysfunction, which is predicted to promote the early development of atherosclerosis and precede plaque formation [40]. The deposition of urate crystals on the vessel wall could cause an inflammatory reaction to then directly injure the vascular intima, ultimately activating the platelet and blood coagulation system. Lastly, hyperuricemia also promotes thrombosis and activates monocyte chemotactic protein-1, an important chemokine in atherosclerosis [2, 41, 42].

The strength of our study is that we began by separating a subset of subjects who were normotensive, without DM, hyperlipidemia, reduced eGFR, or overweight/obese. Despite removing these comorbidities, hyperuricemia remained a risk factor for incident CAD events in crude analyses. While this is likely true for elderly populations, the application of these findings to younger populations is a limitation. Other limitations to our study includes possible selection bias, as all eligible subjects were enrolled from one city. Our study was also limited in that, from 2009 to 2011, we failed to exclude subjects whose eGFR was 60 to 75 mL/min per 1.73 m2, which qualifies as abnormal renal function but not defined as CKD. Failure to exclude these subjects may interfere with analyzing the association between SUA and incident CAD events.

Furthermore, according to the 2013 ESC Guidelines on Stable Angina Pectoris [43], a screening test in asymptomatic individuals without clinical suspicion of coronary artery disease is not recommended. In addition, in a large community-based study of patients with diabetes and no serious diabetes-related complications or hypertension, only 6% of these patients were found to have perfusion defects on exercise thallium scintigraphy, suggesting that the true prevalence of flow-limiting CAD was very low indeed [44]. In our study, all subjects had no comorbidities, which suggested that the prevalence of CAD in these patients may be lower than that in diabetics. Nonetheless, those patients with asymptomatic, but in fact severe CAD, may interfere with the conclusions of the study. Moreover, our analysis only used baseline data, which did not cover the change in SUA levels during the observation period. Lastly, our study may lack generalization to other ethnic populations, as all patients were recruited from China.

The importance of this study is that it shows that the presence of hyperuricemia in elderly adults without comorbidities carries a 2-fold risk for developing incident CAD events within 5 years. Asymptomatic hyperuricemia is a valuable biomarker for predicting the development of incident CAD events. Further prospective research is needed to evaluate whether strategies to reduce SUA over time can prevent this condition.

## MATERIALS AND METHODS

### Study design and study subjects

This study was a large-scale, longitudinal cohort study. The study population was an apparently healthy population because they came to Kangjian Community Health Center to have annual regular health check-up by themselves and also provided a general history for comorbidities. No patients were involved in determining the research objectives or outcome measures, nor were they involved in the design and implementation of the study. This study was conducted according to the principles expressed in the Declaration of Helsinki. The Ethics Committees of Kangjian Community Health Center approved the protocol of this study and waived the need for written informed consent because the data were analyzed anonymously for this observational study.

Subjects (age ≥ 65 years) were enrolled between January 1, 2009 and December 31, 2011. We excluded subjects with hypertension, DM, pre-existing CVD, hyperlipidemia, CKD whose eGFR was < 60 mL/min per 1.73 m^2^, overweight or obesity whose BMI was ≥ 25 kg/m^2^, and hyperuricemia or gout who had medication, irrespective of situation of their drugs used. Baseline demographic and clinical data were collected at the initiation of this study. We used the baseline SUA measurement as the study entry date. The eligible subjects visited the center once every 12 months and were followed until death, loss of follow-up or for 5 years from the initiation of this study. Indications for uric acid-lowering drugs were determined by each patient's physician during the observation period.

### Primary outcome and definitions

The primary outcome of interest was incident CAD events, which included the following: recognized myocardial infarction (MI) and coronary insufficiency (prolonged ischemic chest discomfort associated with transient repolarization abnormality, without criteria for myocardial infarction). Events that were more equivocal, such as unrecognized myocardial infarction and angina pectoris, were not included as CAD events for this analysis. If the patients developed CAD events in any hospital, hospital and physician records were referred to for the diagnosis of CAD events. If CAD events occurred outside a hospital, experts would obtain a consensus about the diagnosis of CAD events after a comprehensive consideration of the history, recent situations, signs, and symptoms before and after CAD events from the patient***’***s medical records in our center and descriptions provided by family members.

Hyperuricemia was defined as > 7.0 mg/dL of SUA in men and ≥ 6.0 mg/dL in women as the standard definition for most studies. BP readings were obtained using an automatic brachial sphygmomanometer (OMRON Corporation, Kyoto, Japan). Two BP examinations were taken after the participant had been seated and resting quietly for > 5 minutes with feet on the ground and back supported. The mean systolic and diastolic BP of each of the participants were calculated from the recorded measurements [12]. BMI was calculated as the weight in kilograms divided by the square of the height in meters. Hypertension was defined as a systolic BP ≥ 140 mmHg or a diastolic BP ≥ 90 mmHg. Patients currently using antihypertensive medications were also classified as positive for hypertension. Patients who reported current use of oral hypoglycemic agents or insulin or who had a clinical diagnosis of diabetes were considered to have diabetes [45]. We defined pre-existing CVD as history of CAD, congestive heart failure (including chronic heart failure long-term use of diuretics), stroke, or peripheral vascular disease. Hyperlipidemia was defined by serum concentrations of cholesterol ≥ 5.7 mmol/L, low density lipoprotein levels ≥ 3.6 mmol/L, or as patients who were currently undergoing treatment with lipid-lowering agents [46]. Individuals who reported smoking of at least one cigarette per day during the year before examination were classified as smoking. Drinking habits were defined as those who consumed more than 20g ethanol per day.

Biochemical parameters, including SUA, hemoglobin, total protein, serum albumin, serum creatinine, total-cholesterol, triglycerides, LDL and HDL were collected every 12 months after this study was initiated. The eGFR levels were calculated by the simplified Modification of Diet in Renal Disease equation [47]. CKD was defined as having an eGFR of < 60 ml/min per 1.73 m^2^.

### Statistical analysis

We divided the eligible subjects into hyperuricemia and normouricemia using baseline SUA level. Data are expressed as mean ± standard derivation or as percent frequency, unless otherwise specified. Comparisons between 2 groups were performed with t tests for normally distributed variables and χ^2^ analyses for categorical data. We used Poisson regression to directly estimate hazards ratio (HR) for incident CAD events between hyperuricemic and normouricemic subjects [48]. Survival was calculated using the Kaplan-Meier method and differences between distributions of survival were assessed by log-rank test. We analyzed the HR of risk for incident CAD events by multivariable Cox regression models. We compared cumulative incidences of incident CAD events over 5 years between hyperuricemia and normouricemia and calculated HR for incident CAD events by crude analysis and after adjustments for age, sex, smoking and drinking habits (Model 1), with the addition of baseline BMI, systolic and diastolic BP (Model 2), and with the addition of eGFR, total-cholesterol, triglycerides, LDL and HDL (Model 3). Furthermore, we calculated cumulative incidences of incident CAD events over 5 years for various levels of SUA, including ≤ 3.0, 3.1 to 4.0, 4.1 to 5.0, 5.1 to 6.0, 6.1 to 7.0, 7.1 to 8.0, and ≥ 8.1 mg/dL. In addition, because the distribution of SUA differed between men and women, multivariable regression analyses were also stratified by sex. The statistically significant level was set α=0.05, and all statistical analyses were 2-sided. All statistical analyses were performed with the SPSS statistics software (IBM SPSS Statistics version 22 for Windows; IBM, New York).

## Abbreviations

BMI: body mass index
BP: blood pressure
CAD: coronary artery disease
CI: confidence index
CKD: chronic kidney disease
CVD: cardiovascular disease
DM: diabetes mellitus
eGFR: estimated glomerular filtration rate
HDL: high density lipoprotein
HR: hazard ratio
LDL: low density lipoprotein
SUA: serum uric acid.

## References

[1] Kim SY, Guevara JP, Kim KM, Choi HK, Heitjan DF, Albert DA (2010). Hyperuricemia and coronary heart disease: a systematic review and meta-analysis. Arthritis Care Res (Hoboken).

[2] Baker JF, Krishnan E, Chen L, Schumacher HR (2005). Serum uric acid and cardiovascular disease: recent developments, and where do they leave us?. Am J Med.

[3] Wannamethee SG (2005). Serum uric acid and risk of coronary heart disease. Curr Pharm Des.

[4] Tseng CH (2004). Independent association of uric acid levels with peripheral arterial disease in Taiwanese patients with Type 2 diabetes. Diabet Med.

[5] Baker JF, Schumacher HR, Krishnan E (2007). Serum uric acid level and risk for peripheral arterial disease: analysis of data from the multiple risk factor intervention trial. Angiology.

[6] Kim SY, De Vera MA, Choi HK (2008). Gout and mortality. Clin Exp Rheumatol.

[7] Feig DI, Kang DH, Johnson RJ (2008). Uric acid and cardiovascular risk. N Engl J Med.

[8] Zhao M, Wang X, He M, Qin X, Tang G, Huo Y, Li J, Fu J, Huang X, Cheng X, Wang B, Hou FF, Sun N (2017). Homocysteine and Stroke Risk: Modifying Effect of Methylenetetrahydrofolate Reductase C677T Polymorphism and Folic Acid Intervention. Stroke.

[9] Mazzali M, Hughes J, Kim YG, Jefferson JA, Kang DH, Gordon KL, Lan HY, Kivlighn S, Johnson RJ (2001). Elevated uric acid increases blood pressure in the rat by a novel crystal-independent mechanism. Hypertension.

[10] Kang DH, Nakagawa T, Feng L, Watanabe S, Han L, Mazzali M, Truong L, Harris R, Johnson RJ (2002). A role for uric acid in the progression of renal disease. J Am Soc Nephrol.

[11] Nakagawa T, Hu H, Zharikov S, Tuttle KR, Short RA, Glushakova O, Ouyang X, Feig DI, Block ER, Herrera-Acosta J, Patel JM, Johnson RJ (2006). A causal role for uric acid in fructose-induced metabolic syndrome. Am J Physiol Renal Physiol.

[12] Kuwabara M, Niwa K, Hisatome I, Nakagawa T, Roncal-Jimenez CA, Andres-Hernando A, Bjornstad P, Jensen T, Sato Y, Milagres T, Garcia G, Ohno M, Lanaspa MA (2017). Asymptomatic Hyperuricemia Without Comorbidities Predicts Cardiometabolic Diseases: Five-Year Japanese Cohort Study. Hypertension.

[13] Johnson RJ, Tuttle KR (2000). Much ado about nothing, or much to do about something? The continuing controversy over the role of uric acid in cardiovascular disease. Hypertension.

[14] Johnson RJ, Kivlighn SD, Kim YG, Suga S, Fogo AB (1999). Reappraisal of the pathogenesis and consequences of hyperuricemia in hypertension, cardiovascular disease, and renal disease. Am J Kidney Dis.

[15] Minino AM, Heron MP, Murphy SL, Kochanek KD (2007). Deaths: final data for 2004. Natl Vital Stat Rep.

[16] Benjamin EJ, Blaha MJ, Chiuve SE, Cushman M, Das SR, Deo R, de Ferranti SD, Floyd J, Fornage M, Gillespie C, Isasi CR, Jimenez MC, Jordan LC (2017). Heart Disease and Stroke Statistics-2017 Update: A Report From the American Heart Association. Circulation.

[17] Gertler MM, Garn SM, Levine SA (1951). Serum uric acid in relation to age and physique in health and in coronary heart disease. Ann Intern Med.

[18] Klein R, Klein BE, Cornoni JC, Maready J, Cassel JC, Tyroler HA (1973). Serum uric acid. Its relationship to coronary heart disease risk factors and cardiovascular disease, Evans County, Georgia. Arch Intern Med.

[19] Brand FN, McGee DL, Kannel WB, Stokes J, Castelli WP (1985). Hyperuricemia as a risk factor of coronary heart disease: The Framingham Study. Am J Epidemiol.

[20] Freedman DS, Williamson DF, Gunter EW, Byers T (1995). Relation of serum uric acid to mortality and ischemic heart disease. The NHANES I Epidemiologic Follow-up Study. Am J Epidemiol.

[21] Wannamethee SG, Shaper AG, Whincup PH (1997). Serum urate and the risk of major coronary heart disease events. Heart.

[22] Culleton BF, Larson MG, Kannel WB, Levy D (1999). Serum uric acid and risk for cardiovascular disease and death: the Framingham Heart Study. Ann Intern Med.

[23] Dobson A (1999). Is raised serum uric acid a cause of cardiovascular disease or death?. Lancet.

[24] Liese AD, Hense HW, Lowel H, Doring A, Tietze M, Keil U (1999). Association of serum uric acid with all-cause and cardiovascular disease mortality and incident myocardial infarction in the MONICA Augsburg cohort. World Health Organization Monitoring Trends and Determinants in Cardiovascular Diseases. Epidemiology.

[25] Weir CJ, Muir SW, Walters MR, Lees KR (2003). Serum urate as an independent predictor of poor outcome and future vascular events after acute stroke. Stroke.

[26] Bos MJ, Koudstaal PJ, Hofman A, Witteman JC, Breteler MM (2006). Uric acid is a risk factor for myocardial infarction and stroke: the Rotterdam study. Stroke.

[27] Burgos J, Cassis S, Kunstmann G, Hernandez A (1991). [Adrenal hemorrhage in newborn infants]. Rev Chil Pediatr.

[28] Sakata K, Hashimoto T, Ueshima H, Okayama A (2001). Absence of an association between serum uric acid and mortality from cardiovascular disease: NIPPON DATA 80, 1980–1994. National Integrated Projects for Prospective Observation of Non-communicable Diseases and its Trend in the Aged. Eur J Epidemiol.

[29] Wheeler JG, Juzwishin KD, Eiriksdottir G, Gudnason V, Danesh J (2005). Serum uric acid and coronary heart disease in 9,458 incident cases and 155,084 controls: prospective study and meta-analysis. PLoS Med.

[30] Zhu Y, Pandya BJ, Choi HK (2012). Comorbidities of gout and hyperuricemia in the US general population: NHANES 2007–2008. Am J Med.

[31] Fang J, Alderman MH (2000). Serum uric acid and cardiovascular mortality the NHANES I epidemiologic follow-up study, 1971–1992. National Health and Nutrition Examination Survey. JAMA.

[32] Sanchez-Lozada LG, Tapia E, Avila-Casado C, Soto V, Franco M, Santamaria J, Nakagawa T, Rodriguez-Iturbe B, Johnson RJ, Herrera-Acosta J (2002). Mild hyperuricemia induces glomerular hypertension in normal rats. Am J Physiol Renal Physiol.

[33] Mazzali M, Kanellis J, Han L, Feng L, Xia YY, Chen Q, Kang DH, Gordon KL, Watanabe S, Nakagawa T, Lan HY, Johnson RJ (2002). Hyperuricemia induces a primary renal arteriolopathy in rats by a blood pressure-independent mechanism. Am J Physiol Renal Physiol.

[34] Dyer AR, Liu K, Walsh M, Kiefe C, Jacobs DR, Bild DE (1999). Ten-year incidence of elevated blood pressure and its predictors: the CARDIA study. Coronary Artery Risk Development in (Young) Adults. J Hum Hypertens.

[35] Jossa F, Farinaro E, Panico S, Krogh V, Celentano E, Galasso R, Mancini M, Trevisan M (1994). Serum uric acid and hypertension: the Olivetti heart study. J Hum Hypertens.

[36] De Scheerder IK, van de Kraay AM, Lamers JM, Koster JF, de Jong JW, Serruys PW (1991). Myocardial malondialdehyde and uric acid release after short-lasting coronary occlusions during coronary angioplasty: potential mechanisms for free radical generation. Am J Cardiol.

[37] Iribarren C, Folsom AR, Eckfeldt JH, McGovern PG, Nieto FJ (1996). Correlates of uric acid and its association with asymptomatic carotid atherosclerosis: the ARIC Study. Atherosclerosis Risk in Communities. Ann Epidemiol.

[38] Gomez M, Vila J, Elosua R, Molina L, Bruguera J, Sala J, Masia R, Covas MI, Marrugat J, Fito M (2014). Relationship of lipid oxidation with subclinical atherosclerosis and 10-year coronary events in general population. Atherosclerosis.

[39] Suarna C, Dean RT, May J, Stocker R (1995). Human atherosclerotic plaque contains both oxidized lipids and relatively large amounts of alpha-tocopherol and ascorbate. Arterioscler Thromb Vasc Biol.

[40] Higgins P, Dawson J, Lees KR, McArthur K, Quinn TJ, Walters MR (2012). Xanthine oxidase inhibition for the treatment of cardiovascular disease: a systematic review and meta-analysis. Cardiovasc Ther.

[41] Kuwano K, Ikeda H, Oda T, Nakayama H, Koga Y, Toshima H, Imaizumi T (1996). Xanthine oxidase mediates cyclic flow variations in a canine model of coronary arterial thrombosis. Am J Physiol.

[42] Visy JM, Le Coz P, Chadefaux B, Fressinaud C, Woimant F, Marquet J, Zittoun J, Visy J, Vallat JM, Haguenau M (1991). Homocystinuria due to 5,10-methylenetetrahydrofolate reductase deficiency revealed by stroke in adult siblings. Neurology.

[43] Montalescot G, Sechtem U, Achenbach S, Andreotti F, Arden C, Budaj A, Bugiardini R, Crea F, Cuisset T, Di Mario C, Ferreira JR, Gersh BJ, Gitt AK (2013). 2013 ESC guidelines on the management of stable coronary artery disease: the Task Force on the management of stable coronary artery disease of the European Society of Cardiology. Eur Heart J.

[44] (1997). Prevalence of unrecognized silent myocardial ischemia and its association with atherosclerotic risk factors in noninsulin-dependent diabetes mellitus. Milan Study on Atherosclerosis and Diabetes (MiSAD) Group. Am J Cardiol.

[45] Bengtsson C, Lapidus L, Stendahl C, Waldenstrom J (1988). Hyperuricaemia and risk of cardiovascular disease and overall death. A 12-year follow-up of participants in the population study of women in Gothenburg, Sweden. Acta Med Scand.

[46] Go AS, Chertow GM, Fan D, McCulloch CE, Hsu CY (2004). Chronic kidney disease and the risks of death, cardiovascular events, and hospitalization. N Engl J Med.

[47] Ito H, Abe M, Mifune M, Oshikiri K, Antoku S, Takeuchi Y, Togane M (2011). Hyperuricemia is independently associated with coronary heart disease and renal dysfunction in patients with type 2 diabetes mellitus. PLoS One.

[48] He F, Wu X, Xia X, Peng F, Huang F, Yu X (2013). Pneumonia and mortality risk in continuous ambulatory peritoneal dialysis patients with diabetic nephropathy. PLoS One.

